# Teacher Punishment Intensity and Parental Trust: A Moderated Moderation Effect Based on CEPS 2013–2014 Survey Data

**DOI:** 10.3390/bs15050608

**Published:** 2025-05-01

**Authors:** Zhen Zhang, Xiaoyu Huang, Yali Zhao, Juan Guo, Chunhui Qi, Guoxiang Zhao

**Affiliations:** 1 Faculty of Education, Henan Normal University, Xinxiang 453007, China; zhangzhenpsy@126.com (Z.Z.); 19100274067@stu.htu.edu.cn (X.H.); 2310283142@stu.htu.edu.cn (Y.Z.); guojuan8533@163.com (J.G.); 2 Faculty of Education, Henan University, Kaifeng 475001, China

**Keywords:** punishment intensity, violation severity, parental trust, teacher gender

## Abstract

Teacher punishment serves as a critical tool not only for fostering the healthy development of adolescents but also for shaping the trust relationship between parents and teachers. Drawing on signaling theory and just deserts theory, this study examines baseline data from the China Education Panel Survey (CEPS 2013–2014) to explore the effects of teacher discipline intensity, student violation severity, and teacher gender on parental trust. The findings indicate the following: (1) There is a significant positive correlation between disciplinary intensity and violation severity, while both exhibit a significant negative correlation with parental trust. Teacher gender also significantly correlates positively with parental trust. (2) Violation severity moderates the negative relationship between teacher discipline intensity and parental trust. Specifically, teacher discipline intensity significantly negatively predicts parental trust under both high- and low-violation conditions, with a more pronounced negative effect under low-violation conditions; (3) For male teachers, there is a significant interaction effect between discipline intensity and violation severity on parental trust, whereas this interaction effect is not observed for female teachers. These results provide valuable insights for teachers in understanding the rationale and methods for implementing appropriate discipline to enhance parental trust.

## 1. Introduction

Mutual trust between teachers and parents serves as the cornerstone for fostering home–school collaboration and parental engagement in education, permeating the entire academic journey of children and adolescents (Hummel et al., 2023a, 2023b; Zhang et al., 2024). Trust acts as a facilitator in home–school relations, playing an essential role not only in initiating, establishing, and sustaining positive interactions but also in promoting the overall development and success of students, classrooms, and schools (Bormann et al., 2021; Niedlich et al., 2021; Shayo et al., 2021). Over the past three decades, the emergence, evolution, and determinants of home–school trust have garnered significant attention across various disciplines, including education (Niedlich et al., 2021), psychology (Shayo et al., 2021), and sociology (Bormann et al., 2021), leading to extensive, in-depth, and systematic research (Rautamies et al., 2021; Santiago et al., 2016; Uitto et al., 2021). As a critical element of home–school trust, parental trust in teachers encompasses the willingness and actions of parents to voluntarily entrust their children to teachers, grounded in their confidence in the teachers’ kindness, reliability, competence, honesty, and openness (Tschannen-Moran & Hoy, 1998). Numerous studies using qualitative methods, questionnaires, and other quantitative techniques have demonstrated that a majority of parents place their trust in their children’s teachers (Huang, 2022; Janssen et al., 2012; Schuster et al., 2025). Furthermore, parental trust is shaped by a variety of individual, family, teacher, and school-related factors (Adams & Christenson, 2000; Bower et al., 2011; Forsyth et al., 2006; Kikas et al., 2011, 2016; Lerkkanen & Pakarinen, 2021).

During interactions between parents and teachers, parents often rely on various social cues—such as teacher gender, professional qualifications, and management style—to determine the extent of their trust in the teacher (Kikas et al., 2011, 2016; Schuster et al., 2025). Among the numerous factors influencing this trust, teacher discipline has consistently garnered significant attention from families, educational institutions, and society at large, as it directly impacts parents’ trust in educators. In 2009, the Chinese Ministry of Education issued the “Regulations on the Work of Headteachers in Primary and Secondary Schools”, explicitly granting teachers the authority to appropriately criticize students as part of their daily educational management responsibilities. Moreover, the Ministry of Education formulated and promulgated the “Rules on Punishment for Primary and Secondary Education (Trial Implementation)”, which officially took effect on 1 March 2021. This policy document defines educational punishment as “an educational act in which schools and teachers manage, discipline, or correct students through prescribed means with the aim of fostering education, thereby encouraging students to reflect upon, comprehend, and rectify their mistakes”. It explicitly clarifies that educational punishment is not punitive in nature but rather a form of educational methodology. The implementation of educational punishment must adhere to the principles of educational value, legality, and appropriateness. In cases where it is deemed necessary, schools and teachers may impose educational punishments on students who exhibit disobedience, disrupt order, engage in improper behavior, pose dangers, or infringe upon the rights and interests of others. Educational punishment is categorized into three levels: general punishment (e.g., verbal reprimands), more severe punishment (e.g., performing public service within the school), and severe punishment (e.g., temporary suspension from school). Some preliminary studies have indicated that schools, teachers, and parents generally concur on the necessity and significance of educational discipline (Qin et al., 2022; Y. Wang et al., 2021).

Utilizing the framework of economic game theory, a substantial body of recent research in behavioral economics has demonstrated that third-party punishment can influence both the trust ratings assigned by observers and the actual behaviors of those administering punishment (Jordan et al., 2016; Salcedo & Jimenez-Leal, 2024; Sun et al., 2023). Furthermore, in business management, L. Wang and Murnighan (2017) found that appropriate disciplinary measures enacted by supervisors can enhance observers’ perceptions of trust and their subsequent behaviors towards those supervisors. Notably, Zhang and Qi (2024) identified a similar effect within school management. However, current research has not thoroughly examined how teacher discipline influences parental trust within the context of home–school interactions. Given that strong parental trust is a critical prerequisite for effective home–school collaboration and mutual support, it is imperative to investigate the impact of teacher disciplinary practices on parental trust in the context of school management.

### 1.1. Disciplinary Intensity and Parental Trust

Educational discipline encompasses the behaviors exhibited by educators in managing, instructing, or correcting students who contravene established rules and regulations, all conducted in a manner aligned with educational objectives (Zhang & Qi, 2024). The primary aim of such disciplinary actions is to facilitate student learning from their mistakes and promote the recognition and rectification of errors. Within the context of school management, educators predominantly employ a system of rewards and punishments to guide student behavior. The implementation of discipline not only affects individual students who infringe upon rules but also has significant spillover effects on their peers and even parents (Sun et al., 2023; L. Wang & Murnighan, 2017; Zhang & Qi, 2024; Zhang et al., 2025a). The intensity of disciplinary actions varies globally, influenced by differing legal norms, ranging from mild verbal reprimands to severe consequences such as expulsion. Signaling theory posits that individuals’ actions and statements can communicate their moral values to observers, thereby shaping the level of interpersonal trust these observers may develop towards them (Connelly et al., 2011; Gintis et al., 2001). In the context of school management, punishment serves as a mechanism to modify students’ compliance behavior, aiming primarily at correction and prevention of deviant behavior rather than retribution for misconduct. However, excessive and severe punishment may project an image of harshness and lack of empathy, thereby diminishing observers’ trust. A substantial body of research indicates that individuals administering punishment are frequently perceived as unpleasant and untrustworthy (Horita, 2010; Kiyonari & Barclay, 2008; Raihani & Bshary, 2019). Furthermore, punitive measures that are excessively harsh, intended to harm, or motivated by self-interest tend to erode bystanders’ trust in the disciplinarian (Spadaro et al., 2023; Sun et al., 2023; L. Wang & Murnighan, 2017; Zhang & Qi, 2024). Consequently, we propose that the intensity of disciplinary actions is negatively associated with parental trust.

### 1.2. Violation Severity as a Moderator

Violation severity pertains to the assessment of the gravity of violations based on factors such as intention, frequency, and consequences of the misconduct (Eriksson et al., 2017; Peterson, 2024). When addressing students who have breached rules and regulations and require disciplinary action, educators must carefully evaluate the appropriateness of punishment. The principle of proportionality in punishment, a key tenet in administrative penalties, serves as an important reference for teachers when implementing disciplinary measures. According to the just deserts theory (JDT), the severity of punishment should be commensurate with the seriousness of the violation (Mooijman & Graham, 2018). Consequently, appropriately measured punishment can enhance interpersonal trust, whereas lenient or disproportionate punishment may undermine it (Peterson, 2024; L. Wang & Murnighan, 2017; Zhang & Qi, 2024). For instance, a study by Zhang and Qi (2024) investigated the impact of disciplinary intensity and violation severity on bystander students’ trust within a school management context, revealing that appropriate disciplinary measures can enhance interpersonal trust among bystander students, whereas inappropriate discipline can diminish it. Concurrently, a meta-analysis concerning leader-employee dynamics indicated that accidental punishment is significantly positively correlated with employees’ trust in their leaders, whereas non-accidental punishment is significantly negatively correlated with trust in leaders (Podsakoff et al., 2006). Consequently, we propose that the severity of students’ violations may positively moderate the negative relationship between the intensity of teachers’ disciplinary actions and parents’ trust.

### 1.3. Teacher Gender as a Second-Order Moderator

In addition to the potential moderating effect of the severity of violations, teacher gender serves as an important social factor that warrants consideration. Social role theory posits that different social norms and cultural expectations shape gender roles. Specifically, male gender roles tend to emphasize the motivational dimension, which is associated with goal achievement and task functionality, including traits such as competitiveness, dominance, and achievement motivation. Conversely, female gender roles often highlight the dimension of inclusiveness, which pertains to relationship maintenance and social functions, encompassing altruistic traits like friendliness, nurturing, and selflessness (Eagly & Wood, 1999). These gender role beliefs not only influence men’s and women’s perceptions and behavioral responses to transgressions (Chawla et al., 2020) but also affect others’ perceptions of their interpersonal trustworthiness. Research indicates that men are more likely than women to perceive violations negatively and impose harsher punishments (Kromer & Bahçekapili, 2010; Zhang et al., 2023), while women exhibit greater trustworthiness and are more likely to gain trust from others (Buchan et al., 2008; Chaudhuri et al., 2013). Consequently, parents may perceive male teachers who enforce discipline as less trustworthy compared to female teachers, particularly when the disciplinary actions are deemed inappropriate. Therefore, we propose that male teachers exert a more significant moderating effect on the negative relationship between the intensity of disciplinary actions and parental trust compared to female teachers.

**Figure 1:**
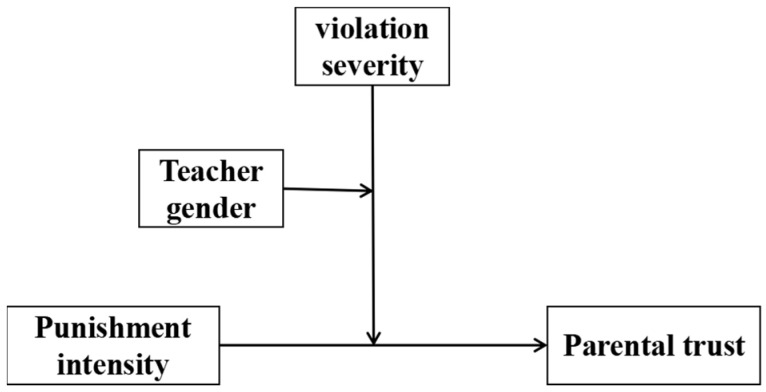
Research model.

In short, this study constructed a moderated moderating effect model to comprehensively examine the effects of teacher discipline intensity, student violation severity, and teacher gender on parents’ trust. The graphical representation of the research model is shown in Figure 1.

## 2. Materials and Methods

### 2.1. Data Sources

The data utilized in this study were sourced from the China Education Panel Survey (CEPS) database. This project, designed and executed by the China Survey and Data Center at Renmin University of China, provides a nationally representative, multi-level dataset. China’s education system shares similarities with the K-12 system in the United States. Specifically, the primary education stage encompasses grades 1 through 6, the secondary education stage includes grades 7 through 9, and the high school stage covers grades 10 through 12. Moreover, both the primary and junior secondary education stages fall under the category of compulsory education, meaning that children are required to complete these two stages of schooling. To date, two waves of data have been released for the academic years 2013–2014 and 2014–2015. The 2013–2014 baseline survey of the China Education Panel Survey was administered to students in grades 7 and 9. Using population average educational attainment and the proportion of floating population as stratification variables, the survey randomly selected 28 county-level units (counties, districts, and cities) across China as sampling sites. The survey was school-based, with 112 schools and 438 classes randomly selected from these units. All students in the selected classes were included in the sample, resulting in a baseline survey that covered approximately 20,000 students. For the purposes of this research, the 2013–2014 baseline survey data were selected, specifically focusing on student questionnaire data that contained no missing values in key indicators. Consequently, the empirical analysis incorporated a sample size of 16,497 individuals. Within this sample, approximately 51% were male, 52% were ninth-grade students, 55% were non-only child, and the average age was 13.49 years. Additionally, about 31% of the participants were residential students. The majority of households were non-mobile and exhibited favorable economic conditions. The study was approved by the Ethics Committee of the Faculty of Education, Henan Normal University.

### 2.2. Variable Selection

#### 2.2.1. Predictor Variable

The primary predictor variable in this study is the intensity of teacher discipline, as reported by students in the student questionnaire. As criticism is a form of general disciplinary action prescribed by Chinese law that teachers can directly administer, and prior literature indicates that criticism serves as a mild yet prevalent method of discipline within Chinese culture (Jin & Yang, 2022; Zhang & Qi, 2024), punishment intensity in this study is defined as the frequency with which teachers criticize students. This construct is measured using two items from the CEPS questionnaire: “The head teacher often criticizes me” and “My parents often receive criticism from the teacher”. Responses to these items are recorded on a four-point Likert scale, with options ranging from 1 (“completely disagree”) to 4 (“completely agree”). A higher score indicates a greater perceived level of disciplinary action. The internal consistency of this measure, as indicated by a Cronbach’s alpha coefficient, is 0.62.

#### 2.2.2. Outcome Variable

The primary outcome variable in this study was the level of trust parents have in teachers, as reported in the parent questionnaire. This was assessed through specific questions such as, “Do you feel that the teacher is responsible for this child?” and “Do you think the teacher will be patient with this child?” Responses were recorded on a five-point Likert scale, where scores ranged from 1 (indicating “not responsible at all/not patient”) to 5 (indicating “very responsible/patient”). Higher scores reflected greater parental trust in teachers. The internal consistency of the scale, as measured by Cronbach’s alpha, was 0.87.

#### 2.2.3. Moderating Variables

The initial moderating variable considered was the severity of infractions as reported by students in the student questionnaire. Similarly, a systematic study shows that lateness, unpunctuality, truancy and skipping classes are relatively common deviant behaviors in middle school management (Crawshaw, 2015). Consequently, this study defines violation severity as the student’s typical frequency of class attendance. This was assessed using two specific items from the CEPS student questionnaire: “I am often late for class” and “I am often absent from class”, each rated on a four-point scale. Higher scores indicate a greater frequency of violations, with an internal consistency coefficient of 0.68. The second moderating variable was the gender of the teacher, as reported by the teacher in the homeroom questionnaire.

#### 2.2.4. Outcome Variables

Drawing on prior research (Forsyth et al., 2006; Kikas et al., 2011, 2016; Lerkkanen & Pakarinen, 2021), it is evident that parental trust is affected by various demographic, familial, class, and school-related factors. Consequently, this study incorporates several control variables, including student gender, age, grade level, and school residence, as well as whether the student is an only child, parental status, family economic status, family mobility, class ranking, the teaching tenure of the homeroom teacher, school type, school location, years of education within the county, and both the category and location of the county (see Table 1). To mitigate the influence of parental status, the analysis is restricted to data pertaining to biological parents only.

### 2.3. Data Analysis

SPSS24.0 software and PROCESS macros were used for data processing. The test idea is as follows: First, descriptive statistics and correlation analysis are carried out for each variable. Secondly, Model 1 in the PROCESS program was used to test the adjustment effect by extracting the bootstrap 95% confidence interval estimated by 5000 samples. Finally, Model 3 in the PROCESS program was used to test the adjusted adjustment effect by extracting the bootstrap 95% confidence interval estimated by 5000 samples.

## 3. Results

### 3.1. Assessment of Common Method Bias

The assessment of common method bias was conducted using Harman’s single-factor test. The analysis revealed the extraction of nine factors with eigenvalues exceeding 1, without rotation. The first factor accounted for 14.44% of the variance, which is below the 40% threshold, suggesting the absence of significant common method bias (Podsakoff et al., 2003).

### 3.2. Descriptive Statistics and Correlation Analysis

A comprehensive statistical analysis was performed to examine the relationships among disciplinary intensity, violation severity, teacher gender, and parental trust (see Table 2). The findings indicate a significant positive correlation between disciplinary intensity and violation severity, as well as a significant negative correlation between disciplinary intensity and parental trust. Additionally, violation severity exhibited a negative correlation with both teacher gender and parental trust. Furthermore, a significant positive correlation was identified between teacher gender and parental trust. Finally, the Skewness and Kurtosis of parental trust were −0.87 and 0.56, respectively, indicating values close to those of a normal distribution. The variance inflation factor (VIF) for all predictors was below 1.17, and the tolerance levels for all predictors were above 0.85, indicating the absence of significant multicollinearity issues.

### 3.3. Moderation Effect Analysis

**Figure 2:**
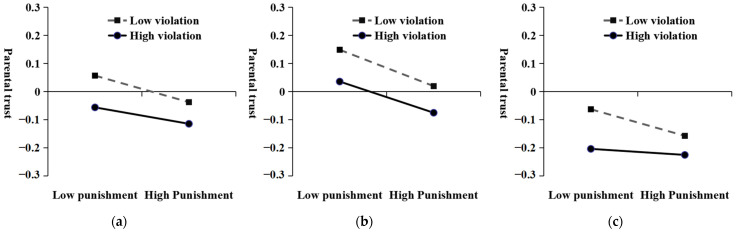
The moderating role of violation severity in the relation between punishment intensity and parental trust for all data (**a**), female teachers (**b**) and male teachers (**c**).

First, Model 1 from the SPSS Process plug-in was employed to examine the moderating effects of disciplinary intensity and violation severity on parental trust. Drawing upon the methodology of Lu et al. (2022), four models for regulatory effect analysis were incrementally integrated with covariates at various levels through hierarchical modeling strategies. Firstly, Model 1-1 serves as a baseline model, concentrating solely on the moderating effects of disciplinary intensity and violation severity on parental trust, thereby establishing a reference point. Secondly, Model 1-2 incorporates five fundamental demographic characteristics of students—gender, age, grade, residence, and only-child status—to control for basic individual attributes. Thirdly, Model 1-3 further includes three familial statistical characteristics: parental status, mobility, and family economic status, to account for economic factors at the family level. Finally, Model 1-4 integrates additional variables such as class ranking, gender of the head teacher, teaching experience of the head teacher, type and location of the school, years of education in the county, and both category and location of the county. These variables control for class, school, and regional factors, thereby providing a comprehensive analysis framework. Taking the most conservative and rigorous model 1-4 as an example (see Table 3), after controlling the variables of personal characteristics, family characteristics, class characteristics, school characteristics and regional characteristics, disciplinary intensity can negatively predict parental trust (*β* = −0.06, *t* = −7.24, *p* < 0.01). Violation severity negatively predicted parental trust (*β* = −0.08, *t* = −7.12, *p* < 0.01). The interaction terms between disciplinary intensity and severity of violation were significant (*β* = 0.02, *t* = 3.05, *p* < 0.01), indicating that the severity of violation had a significant moderating effect between disciplinary intensity and parents’ trust. In order to clarify more clearly the moderating effects of disciplinary intensity and violation severity, we conducted a simple slope test using the point-selection method. The results of point selection method showed that disciplinary intensity in the low-severity group could significantly negatively predict parental trust (*β* = −0.07, *t* = −7.54, *p* < 0.01). In the high-severity group, the severity of discipline also negatively predicted parental trust (*β* = −0.05, *t* = −5.61, *p* < 0.01), but the slope was significantly weakened (see Figure 2a).

Further, to examine the moderating effects of disciplinary intensity, violation severity, and teacher gender on parental trust, we employed Model 3 from the SPSS Process plug-in. Our analysis followed a systematic modeling approach, progressively incorporating various covariates across four moderated effect analysis models. Initially, Model 2-1 served as a baseline, focusing solely on the moderating effects of disciplinary intensity, violation severity, and teacher gender on parental trust. Subsequently, Model 2-2 integrated basic demographic characteristics to control for individual-level variables. Model 2-3 expanded the analysis by including household statistical characteristics, thereby accounting for household economic factors. Finally, Model 2-4 incorporated additional variables to control for class, school, and district-level influences, thus providing the most conservative estimates of the effects. The results of the final analysis indicated that the interaction terms for disciplinary intensity, violation severity, and teacher gender remained statistically significant across all models. Taking the most conservative and rigorous model 2-4 as an example (see Table 3), after controlling for all additional variables, the interaction terms of disciplinary intensity, violation severity and class teacher gender were significant (*β* = −0.01, *t* = −2.15, *p* < 0.05). Further analysis showed that when the teacher was female, the interaction between disciplinary intensity and violation severity was not significant (*β* = 0.01, *F*(1, 16,474) = 1.40, *p* > 0.05). Specifically, disciplinary intensity can significantly negatively predict parents’ trust attitude at both a high severity of violation (*β* = −0.06, *t* = −5.96, *p* < 0.01) and low severity of violation (*β* = −0.07, *t* = −6.79, *p* < 0.01). However, there is no significant difference in slope between the two (see Figure 2b). On the contrary, when the teacher was male, the interaction between disciplinary intensity and violation severity was significant (*β* = 0.03, *F*(1, 16,474) = 13.00, *p* < 0.01). Specifically, the disciplinary intensity can significantly negatively predict the parents’ trust when the severity of violation is low (*β* = −0.05, *t* = −3.63, *p* < 0.01). However, it was not possible to predict parents’ trusting attitude when the severity of violation was high (*β* = −0.02, *t* = −1.40, *p* > 0.05) (see Figure 2c).

## 4. Discussion

The findings of the present study indicate that disciplinary intensity serves as a significant negative predictor of parental trust. Furthermore, the severity of violations moderates the relationship between disciplinary intensity and parental trust, with this moderating effect being contingent upon the gender of the disciplining teacher. Specifically, when the disciplining teacher is male, the severity of the violation influences the relationship between disciplinary intensity and parental trust.

First, the findings demonstrate that teacher disciplinary intensity significantly and negatively predicts parental trust. This result aligns with signal transmission theory, which posits that the intensity of disciplinary actions influences the trust placed in the disciplinarian (Gintis et al., 2001). Consistent with prior research (Sun et al., 2023; Zhang & Qi, 2024), this outcome can be attributed to the perceived lack of warmth associated with increased discipline. The primary objective of teachers’ disciplinary measures is to correct and prevent misbehavior, not to retaliate against or harm students. Excessive punishment can foster a perception of indifference among teachers and diminish the warmth parents perceive from them. Qualitative studies on parent-teacher trust have identified teacher warmth as a crucial trait for establishing healthy relationships between parents and teachers (Huang, 2022; Schuster et al., 2025). Therefore, greater disciplinary severity correlates with lower parental trust in the disciplining teacher. The spillover effect of teacher punishment on parental trust extends the scope of educational management and provides empirical support for home–school collaboration.

Second, the results indicate that the severity of student violations moderates the relationship between disciplinary intensity and parental trust. Specifically, both high- and low-severity groups show that disciplinary intensity significantly and negatively predicts parental trust, although the effect is slightly weaker in the low-severity group. As illustrated in Figure 2a, parental trust remains positive only when teachers impose mild punishments on students with minor violations; otherwise, it is negative. This finding supports the Just Deserts Theory, which posits that the severity of punishment should be proportional to the severity of the violation (Mooijman & Graham, 2018). Otherwise, it may lead to suspicion and distrust from both bystanders and offenders. Numerous studies using economic game tasks and management scenarios have demonstrated that proportionate punishment can enhance bystanders’ trust in the disciplinarian (L. Wang & Murnighan, 2017; Zhang & Qi, 2024). For instance, Zhang and Qi (2024) found that observing teachers’ fair punishment of rule-breaking students significantly improves students’ credibility judgments of those teachers. When violations are severe, the negative impact of disciplinary intensity on parental trust diminishes, possibly because more serious violations raise the threshold for acceptable disciplinary intensity. However, frequent punishments for students who exhibit serious misconduct can significantly erode parental trust in teachers.

Finally, this study reveals that the aforementioned moderating effects are influenced by the gender of the teacher. Specifically, when female teachers discipline, the severity of violations does not moderate the relationship between disciplinary intensity and parental trust. Conversely, male teachers’ disciplinary intensity for minor violations significantly and negatively predicts parental trust, while their intensity for severe violations has no significant effect. This result aligns with the social role theory of gender (Eagly & Wood, 1999), a critical theoretical framework for comprehending gender differences. Social role theory posits that society holds distinct expectation, cognition, and stereotype regarding gender role, which collectively form the basis of social gender role. Specifically, societal norms suggest that male individuals should emphasize rationality, competition, indifference, and achievement, whereas female individuals are expected to prioritize sensibility, cooperation, care, and warmth. Furthermore, individuals of different genders tend to adhere to social norms and expectations, striving to exhibit attitudes and behaviors consistent with these expectations, thereby influencing interpersonal judgments and relationships (Eagly, 2009). On one hand, certain scholars have demonstrated that parental trust is more readily influenced by teachers’ kindness and warmth (Huang, 2022; Schuster et al., 2025). This finding resonates with the current research results, indicating that female teachers elicit greater parental trust compared to male teachers, potentially due to their perceived higher levels of kindness and care, which facilitate the establishment of reliable and trusting interpersonal relationships. On the other hand, these social role expectations significantly impact the cognitive, emotional, and behavioral responses of both men and women in cases of breaches. Extensive studies reveal that men perceive transgressions more negatively than women, experience more intense negative emotions, and are more inclined to impose harsher punishments (Kromer & Bahçekapili, 2010; Balafoutas & Nikiforakis, 2012; Rodriguez-Ruiz et al., 2019; Zhang et al., 2023). Additionally, such role expectations may alter parents’ perceptions of discipline enacted by male and female teachers, influencing their interpersonal trust in disciplinary teachers. Parents are more likely to perceive discipline from female teachers as friendly and pro-social, aimed at fostering student development and growth, thus enhancing their willingness to trust female teachers. Conversely, the same disciplinary actions implemented by male teachers might be attributed to selfish or skill-driven motivations, reflecting a pursuit of status, control, and achievement, ultimately leading to reduced trust in male teachers. Previous studies have shown that punishments perceived as harmful or self-serving reduce bystander trust in the disciplinarian (Spadaro et al., 2023; Sun et al., 2023). Parents tend to perceive female teachers as warmer and more caring, thus showing higher levels of trust. In contrast, parents may attribute male teachers’ disciplinary actions to self-interested motives such as pursuing grades, rankings, or reputation.

In addition to the control and moderating variables emphasized in current research, unobserved variables such as teacher–student relationships may exert significant influence on both disciplinary actions and parental trust. For instance, Zhang et al. (2025b), drawing on data from the China Education Panel Survey, demonstrated a bidirectional predictive relationship between teacher–student relationships and parents’ perception of teacher care, which enhances interpersonal trust in teachers. Simultaneously, some studies have preliminarily confirmed that group relationship could moderate the impact of punishment on bystander trust (Sun et al., 2023; Zhang & Qi, 2024). Specifically, punishment directed at in-group violators tends to weaken bystanders’ interpersonal trust in the disciplinarian. For example, Zhang and Qi (2024) conducted situational experiments manipulating teacher discipline intensity and teacher–student group relationships, revealing that when teachers impose severe discipline on students with close relationships, observers’ interpersonal trust in teachers diminishes significantly. Although the current research has neither tested nor controlled for teacher–student relationships, the moderating effect of such group dynamics is likely applicable within the context of home–school interactions. In essence, when teacher–student relationships are positive, parents anticipate greater tolerance from teachers regarding their children’s mistakes; thus, unexpectedly harsh discipline may reduce parental interpersonal trust in teachers.

## 5. Practical Implications

To our knowledge, this study is the first to demonstrate the spillover effect of teacher discipline on parental trust. The findings have important theoretical and practical implications for home–school collaboration. Educational punishment constitutes a profoundly sensitive and pervasive challenge in school environments. Establishing appropriate parameters for educational discipline, safeguarding students’ legal rights, maintaining teachers’ professional authority, and resolving educators’ reluctance or inability to address behavioral issues have persisted as fundamental concerns in compulsory education systems. Within this framework, the dual imperatives of operationalizing pedagogically sound disciplinary practices and cultivating parental confidence require educators to balance humanistic considerations with educational efficacy. This approach facilitates collaborative partnerships among educational stakeholders while advancing students’ holistic development.

Primary among implementation considerations is the intentionality of disciplinary measures. As a pedagogical tool within institutional governance structures, educational discipline should be purposefully designed to facilitate corrective learning rather than punitive retribution. Educators must therefore ensure that disciplinary interventions primarily serve preventive functions, consciously avoiding measures that inflict undue psychological or physical distress. In other words, teachers need to keep in mind that discipline is for the better development and growth of students.

Equally crucial is maintaining proportionality in disciplinary responses. As a critical guideline in various fields such as law, ethics, and policy-making, the principle of proportionality also offers substantive guidance for educational practitioners. Retributive justice theory posits that disciplinary severity must correlate with both the objective gravity of misconduct and subjective culpability factors (Mooijman & Graham, 2018). Objective assessment requires evaluation of the violation’s societal impact, including its nature, contextual circumstances, and measurable consequences. Concurrently, subjective analysis must consider the offender’s intentionality (e.g., premeditated versus accidental, benevolent versus malicious motivations). Consequently, educators should employ standardized evaluative frameworks that systematically integrate these dimensions, ensuring legally compliant and ethically proportionate disciplinary determinations.

Ultimately, the implementation process must foreground disciplinary pedagogy’s rehabilitative essence. Philosophically grounded in care ethics, this paradigm emphasizes nurturing supportive ecosystems that connect institutional and domestic spheres (Noddings, 2006). Given that disciplinary interventions fundamentally aim at behavioral remediation and moral education, practitioners must maintain acute sensitivity to stakeholders’ perceptions. Only through consensually validated, care-informed disciplinary practices can educators achieve sustainable behavioral modification and pedagogical objectives.

## 6. Limitations

Similar to other studies, this research has limitations. First, given that the current study utilized cross-sectional questionnaire data, establishing causal inferences from this research design remains inherently limited. Although a significant association between teacher disciplinary intensity and parental trust was observed, the absence of temporal precedence precludes definitive causal conclusions. Subsequent investigations could adopt longitudinal methodologies to delineate temporal dynamics and assess sustained impacts of disciplinary approaches on trust. Furthermore, controlled experimental paradigms systematically manipulating infraction scenarios and disciplinary responses may elucidate causal linkages between these constructs.

Second, the reliance on self-reported measures introduces potential response biases. Social desirability tendencies may compel participants to report elevated trust levels incongruent with their actual perceptions of disciplinary rigor. Such measurement artifacts could artificially inflate trust metrics. To mitigate this limitation, multimethod assessments incorporating behavioral proxies—such as parental engagement in school governance or endorsement of teacher recommendations—could yield more ecologically valid trust indicators.

Third, the study’s exclusive focus on secondary education populations constrains the generalizability of findings. Given that pedagogical disciplinary practices span diverse developmental stages (e.g., primary education and tertiary institutions), future replication studies should incorporate stratified samples across educational tiers. This expansion would enhance the external validity of the observed relationships and facilitate cross-contextual comparative analyses.

## 7. Conclusions

The current study demonstrates that teacher discipline intensity negatively predicts parental trust. Moreover, the severity of student violations positively moderates this relationship. Importantly, this moderating effect is observed only in male teachers, not in female teachers. These findings contribute to understanding the spillover effect of teacher discipline on parental trust and offer practical guidance for improving home–school collaboration.

## Figures and Tables

**Table 1:** The explanatory and descriptive statistics of control variables in data analysis.

Type	Variable Name	Items	Variable Description	M	SD
Predictor variable	Punishment intensity	Class teacher often criticizes me	1 = Strongly disagree; 2 = disagree; 3 = Agree; 4 = Strongly agree	1.51	0.65
		My parents often receive criticism about me from the teacher			
Outcome variable	Parental trust	Do you think the teacher is responsible for your child?	1 = Not at all; 2 = Not very; 3 = Average; 4 = Quite; 5 = Very	4.26	0.74
		Do you think the teacher is patient with your child?			
Moderator variable	Violation severity	I often arrive late	1 = Strongly disagree; 2 = disagree; 3 = Agree; 4 = Strongly agree	1.17	0.45
		I often skip classes			
	Teacher gender	Teacher gender	1 = Male; 2 = Female	1.64	0.48
Control variable	Individual characteristics	Student gender	0 = Female; 1 = Male	0.51	0.50
		Student age	Age at the time of the survey	13.49	1.22
		Student grade	0 = grade 7; 1 = grade 9	0.47	0.50
		Boarding status	0 = Not boarding; 1 = boarding	0.31	0.46
		Only child status	1 = Only child; 2 = Not an only child	1.55	0.50
	Family characteristics	Parental identity	1 = Biological father; 2 = Biological mother	1.53	0.50
		Family mobility status	1 = Non-mobile; 2 = Intra-provincial mobile; 3 = Inter-provincial mobile	1.28	0.63
		Family economic status	1 = Difficult; 2 = Medium; 3 = Rich	1.86	0.49
	Class Characteristics	Teacher’s teaching experience	1 = Less than 10 years; 2 = 10–19 years; 3 = More than 20 years	2.07	0.72
		Class ranking	1 = Lower-middle; 2 = Middle; 3 = Best	1.94	0.53
	School Characteristics	School type	1 = Public; 2 = Private	1.94	0.86
		School location	1 = County town; 2 = Urban-rural fringe; 3 = Rural township	1.07	0.25
	Regional Characteristics	County location	1 = East; 2 = Central; 3 = West	1.67	0.83
		County category	1 = Direct-controlled municipality; 2 = Provincial capital; 3 = Prefecture-level city; 4 = County-level	2.92	1.14
		County education levels	1 = Low; 2 = Medium; 3 = High	1.98	0.85

**Table 2:** Descriptive statistics and correlations matrix of all variables (*N* = 16,497).

Variables	M	SD	1	2	3	4
1. Punishment intensity	1.51	0.65	1.00			
2. Violation severity	1.17	0.45	0.38 **	1.00		
3. Teacher gender	1.64	0.48	−0.01	−0.05 **	1.00	
Parental trust	4.26	0.74	−0.09 **	−0.10 **	0.14 *	1.00

Note: Gender was coded as binary variable (1 = male and 2 = female), * *p* < 0.05, ** *p* < 0.01.

**Table 3:** Testing the Moderated Moderation Effect (*N* = 16,497).

Variables	Parent Trust				Parent Trust			
	Model 1-1	Model 1-2	Model 1-3	Model 1-4	Model 2-1	Model 2-2	Model 2-3	Model 2-4
Punishment intensity (A)	−0.07 **	−0.07 **	−0.06 **	−0.06 **	−0.07 **	−0.07 **	−0.07 **	−0.06 **
Violation severity (B)	−0.11 **	−0.08 **	−0.08 **	−0.08 **	−0.10 **	−0.08 **	−0.08 **	−0.08 **
Teacher’s gender (C)					0.14 **	0.12 **	0.11 **	0.09 **
A × B	0.02 **	0.02 **	0.02 **	0.02 **	0.02 **	0.02 **	0.02 **	0.02 **
A × C					−0.01	−0.01	−0.01	−0.01
B × C					0.01	<0.01	<0.01	<0.01
A × B × C					−0.01 *	−0.01 *	−0.01 *	−0.01 *
*R* ^2^	0.01	0.05	0.06	0.08	0.03	0.06	0.07	0.08
*F*	82.74 **	107.39 **	87.81 **	72.56 **	85.29 **	91.53 **	79.07 **	63.40 **

Note: Gender was coded as binary variable (1 = male and 2 = female), * *p* < 0.05, ** *p* < 0.01.

## Data Availability

The raw data supporting the conclusions of this article will be made available by the authors on request.
